# Nutritional evaluation, antioxidant studies and quantification of poly phenolics, in *Roscoea purpurea* tubers

**DOI:** 10.1186/s13104-015-1290-x

**Published:** 2015-07-30

**Authors:** Ankita Misra, Sharad Srivastava, Shikhar Verma, Ajay Kumar Singh Rawat

**Affiliations:** Pharmacognosy and Ethanopharmacology Division, CSIR-National Botanical Research Institute, Lucknow, 226001 India

**Keywords:** *Roscoea purpurea*, Nutritional characterization, HPTLC, Invitro assays, Poly phenolics

## Abstract

**Background:**

*Roscoea purpurea* (Zingiberaceae) is commonly known as “kakoli”. Traditionally, various parts like leaves, roots and flower etc. are used for the treatment of diabetic, hypertension, diarrhea, fever, inflammation etc. In Nepal tubers are boiled for edible purpose and also used in traditional veterinary medicine. The study aims for nutritional characterization, chemical profiling of *R. purpurea* (tubers) methanol extract (RPE) along with evaluation of its anti-oxidant activity. Physicochemical and nutritional content were estimated as per standard protocols. Chemical profiling of markers includes method optimization, identification & quantification of bioactive poly phenolics through HPTLC. Anti oxidant potential RPE was analyzed via. Total phenolics (TPC), total flavonoids (TFC), reducing power assay, DPPH and β-carotene bleaching model.

**Results:**

Physicochemical and nutritional standards were established. Kaempferol (0.30%), vanillic acid (0.27%), protocatechuic (0.14%), syringic (0.80%) and ferulic acid (0.05%) were identified and then quantified. TPC and TFC content were found to be 7.10 ± 0.115 and 6.10 ± 0.055%, reducing power of extract also increases linearly (r^2^ = 0.946) with concentration, similar to standards. IC_50_ value of extract in DPPH and β-carotene bleaching model was observed at 810.66 ± 1.154 and 600.66 ± 1.154 µg/ml, which is significantly different from standards (p < 0.05). Although there is a positive, significant correlation between the phenolic and flavonoid content with anti oxidant activity of extract.

**Conclusion:**

Thus, study will authenticates the identity, utility of herb as nutrient supplement and an important medicinal plant having promising pharmacological activities for further elaborated/extended investigation work.

## Background

*Roscoea purpurea* synonymously known as *Roscoea procera* (Wall.) is a perennial herb belonging to family Zingiberaceae. The specie is locally renowned as kakoli, red gukhra, dhawanksholika, karnika, ksheera, madhura, shukla, svadumansi, vayasoli and vaysasha etc. and is native of Nepal. *R. purpurea* is abundantly available in Himalayas also; on steep, grassy hill sides, damp gullies and stony slopes. Like all members of the genus *Roscoea*, it dies back each year to short vertical rhizomes which are attached to the tuberous roots; the most utilized part. When growth begins again, “pseudo stems” are produced: structures which resemble stems but are actually formed from the tightly wrapped bases (sheaths) of its leaves. *R.* *purpurea* can grow to over 50 cm tall, with wide leaves and a stout pseudo-stem. The leaf sheaths are pale green or may have a dark reddish-purple tinge. *R. purpurea* is cultivated as an ornamental plant, although in northern India fleshy roots are traditionally used for making a tonic to treat malaria and urinary infection. In ethnobotanical practice, the various plants parts like leaves, roots and flower etc. are used for the treatment of diabetic, hypertension, diarrhea, fever, inflammation etc. In Nepal tubers are boiled for edible purpose and also used in traditional veterinary medicine [1, 2]. Tubers of *Roscoea* are major constituent of polyherbal Ayurvedic formulation, “Ashtavarga”, which according to Nikhandu Samhita and Indian Metria Medica is like chawanprash having, anti-oxidant, anti ageing effect and elevates overall health status of a well being [3].

Tubers of *R. purpurea* exhibit immuno-modulatory [4] and antidiabetic activity [5]. Botanical studies on tubers showed the presence of 10–12 layered cork, below the cork phellogen layer is present; cortex consisting of oval to elongated, thin walled, parenchymatous cells filled with abundant, simple, ovoid to ellipsoidal starch grains, followed to vascular bundles composed of usual elements [6]. Inspite of all this available literature, there exist a lot of confusion regarding the authenticity and identity of species and the concrete data on physico-chemical characterization, chemical profiling and nutritional potential is still lacking. Till date no work has yet been carried out on identification and quantification of phenolics in species, neither the antioxidant nor antimicrobial potential of tuber had been evaluated. Hence in the present study an attempt was made with the objectives, to identify the various physico-chemical standards of the species, to validate the edible use of *R. purpurea* tuber through quantification of its nutritional contents, chemical profiling, quantification and method optimization for identification of bioactive polyphenolics and evaluation of its anti oxidant potential.

## Methods

### Reagents

Ascorbic acid (>97%), quercetin (>97%), rutin (>99%), BHT (Butylated hydroxy toluene, >98%), 1-1-diphenyl-2-pic-rylhydrazyl (DPPH), Linoleic acid (>98%) and β-carotene (>95%) were purchased from Sigma-Aldrich. All the solvents and chemicals (AR grade) are obtained from SD Fine Chemicals, Mumbai, India.

### Plant material

Fresh tubers of *Roscoea purpurea* were collected in the month of October–November from the nearby area of Kempti fall, Mussoorie, Uttrakhand (India). Tuber sample was authenticated and voucher specimen (LWG no. 254028) was deposited in herbarium repository of CSIR-National Botanical Research Institute. Collected sample was washed, shade dried and powdered for further studies.

### Physicochemical characterization

Various physico chemical values viz. Moisture content, total ash, water soluble ash, acid insoluble ash and extractive values (hexane, alcohol and water soluble extractives) were evaluated. Sample (powder) was also qualitatively screened to indentify the presence of various phytochemicals [7].

### Nutritional characterization

The percentage of various metabolites i.e. oil [7], sugar and starch [8], phenolics [9], flavonoids [10], crude alkaloid [11], total protein [12] and crude fiber [13] present in tubers were determined as per standard procedures.

### Extract preparation

The dried, chopped tubers of *R. purpurea* were grinded using lab grinder and the powder obtained was passed through 40 mesh (up to 500 mm) sieve. About 100 g was defatted with petroleum ether and then treated with methanol (ethanol stabilized) through soxhlation, till complete exhaustion of sample (7 days; 27 ± 2°C). The pooled extracts were filtered through Whatman no. 1 filter paper and concentrated in rotary evaporation at 50°C under reduced pressure (40 mbar). The concentrated extracts (RPE) were finally lyophilized and quantified.

### Chemical profiling, method optimization, identification and quantification through HPTLC

Chemical profiling and method optimization for evaluation of polyphenolics was performed on 20 cm × 10 cm TLC aluminum pre-coated plates with 200 nm layer thickness of silica gel 60 F_254_ (sd. Finechem. Ltd, Mumbai, India). Tracks (standard and sample) were applied as 6 mm band width using Camag 100 micro liter sample syringes (Hamilton, Switzerland) with a Linomat 5 applicator (Camag, Switzerland) under a flow of N_2_ gas. The Linear ascending development was carried out with Toluene: Ethyl acetate: Formic acid [6:3:1 v/v] as a mobile phase in a Camag glass twin trough chamber. The chamber was previously saturated with mobile phase vapors for 10 min at room temperature (25 ± 2°C) and plates were developed at distance of approximately 80 mm from the point of application (total length run by mobile phase). After development, plates were dried for 30 min and scanning was per formed using Camag TLC Scanner 3 at from 200 to 700 nm in UV absorbance mode for range of flavonoids, operated by win CATS Software (version 3.2.1). The slit dimensions were 4 mm × 0.45 mm and the scanning speed was 100 mm/s. Quantification was performed using peak area with linear regression of amount (ng/band). In the employed experimental condition, the HPTLC method includes evaluation of the following performance parameters also such as linearity, Limit of detection, limit of quantification according to the guidelines [14].

### Determination of polyphenols

Total phenolic content was calculated in terms of mg/gm GAE (Gallic Acid Equivalent) based on calibration curve of Gallic acid as standard [9] and total flavonoid was depicted in terms of mg/gm of QE (Quercetin Equivalent) [10].

### Anti oxidant activity

#### Ferric reducing power assay

The RPE (0.2–1.0 mg/ml) in distilled water were mixed with 2.5 mL of phosphate buffer (0.2 mol/L, pH 6.6) and 2.5 mL of 1% (w/v) potassium ferricyanide. The mixture was incubated at 50°C for 20 min. Following this, 2.5 mL of 10% (w/v) Trichloroacetic acid was added and the mixture was then centrifuged at 800 rpm for 10 min. A 2.5 mL aliquot of supernatant was mixed with 2.5 mL of distilled water and 0.5 mL of 0.1% (w/v) FeCl_3_; the absorbance of the mixture was read at 700 nm [15].

#### DPPH radical scavenging assay

The effect of RPE on DPPH radical was estimated by using the proposed method [16]. Ascorbic acid, Quercetin, Rutin and BHT were used as reference samples (0.1 mg/ml) and the ability of standard/RPE to scavenge DPPH radical was calculated by following equation.

$$\begin{aligned} &{\text{DPPH radical scavenging activity}}\left( \% \right) \\ &= \left( {{\text{Abs}}_{\text{control}} {-}{\text{Abs}}_{\text{sample}} } \right) \times 100/\text{A}{\text{bs}}_{\text{control}} \end{aligned}$$

where, Abs_control_ is the abssorbance of DPPH radical + methanol and Abs_sample_ is the absorbance of DPPH radical +RPE/standard.

#### β-carotene-linoleate assay

The activity of RPE against β-carotene-linoleate was estimated without any modification in standard method [17]. The antioxidant activity (AA) of the RPE was evaluated in terms of bleaching of the β-carotene using the following formula:

$${\text{AA }}\left( \% \right) \, = \, \left[ { 1- \left( {{\text{A}}^{0} - {\text{A}}^{\text{t}} } \right)} \right] \times 100/\left[ { 1- \left( {{\text{A}}_{0}^{0} - {\text{A}}_{t}^{t} } \right)} \right]$$

where A^0^, A_0_^0^ and A^t^, A_t_^0^ are the absorbance values measured at zero time and after incubation for 60 min in the RPE and control, respectively.

### Statistical analysis

Results were expressed as mean ± SD. Linear regressions analysis was carried out for standards to calculate total polyphenols content and graph pad prism 5 software was used to calculate the IC_50_ values. One-way ANOVA followed by student’s t test (p < 0.01) was used to find the significance of standard and sample in anti-oxidant activity. Pearson correlation coefficient for phenolic and flavonoids with IC_50_ value of DPPH and β-Carotene assays were also employed.

## Results

### Estimation of physicochemical and nutritional characters

Moisture content of tuber was 3.22%. Total ash was found to be 5.62%, indicating the presence of in-organic content of the sample, whereas water soluble and acid insoluble ash was 3.95 and 0.30% respectively. Extractive values by cold maceration of tuber’s reveals that water soluble extractive was found to be maximum, followed by alcohol and hexane soluble extractives i.e. 8.66, 6.33 and 5.0% respectively. These extractive values are primarily useful for determination of exhausted or adulterated drug and was found to be within the limits (Table 1) [6]. Phytochemical screening of powder reveals the presence of carbohydrates, proteins, phenolics, flavonoids, alkaloids, glycosides, tannin and saponins.

**Table 1:** Physico chemical values of *R. purpurea* tuber

Parameter^a^	Value (%)
Moisture content	3.22 ± 0.01
Hexane soluble extractive	5.0 ± 0.01
Alcohol soluble extractive	6.33 ± 0.005
Water soluble extractive	8.66 ± 0.01
Total ash	5.62 ± 0.01
Acid insoluble ash	0.3 ± 0.005
Water soluble ash	3.95 ± 0.01

^a^Values are mean ± SD, n = 3.

Quantification of nutritional content (Table 2) in species reveals that fiber was found to be maximum (28.1%), followed to oil, protein, alkaloid, starch, phenolics, flavonoids and sugar content i.e. 3.5, 3.46, 2.3, 0.84, 0.71, 0.61 and 0.29% respectively. Total carbohydrate and gross energy was 82.7% and 1566.2 kg/100 g dry matter [18].

**Table 2:** Nutritional characterization of *R. purpurea* tuber

Nutritional parameters	Values (%)
Sugar	0.29 ± 0.01
Starch	0.84 ± 0.01
Phenolics	0.71 ± 0.005
Flavonoid	0.6 ± 0.01
Protein	3.46 ± 0.01
Alkaloid	2.3 ± 0.01
Oil	3.5 ± 0.005
Fibre	28.1 ± 0.01

Values are mean ± SD, n = 3.

### Identification and quantification of chemical markers in extract

**Fig. 1:**
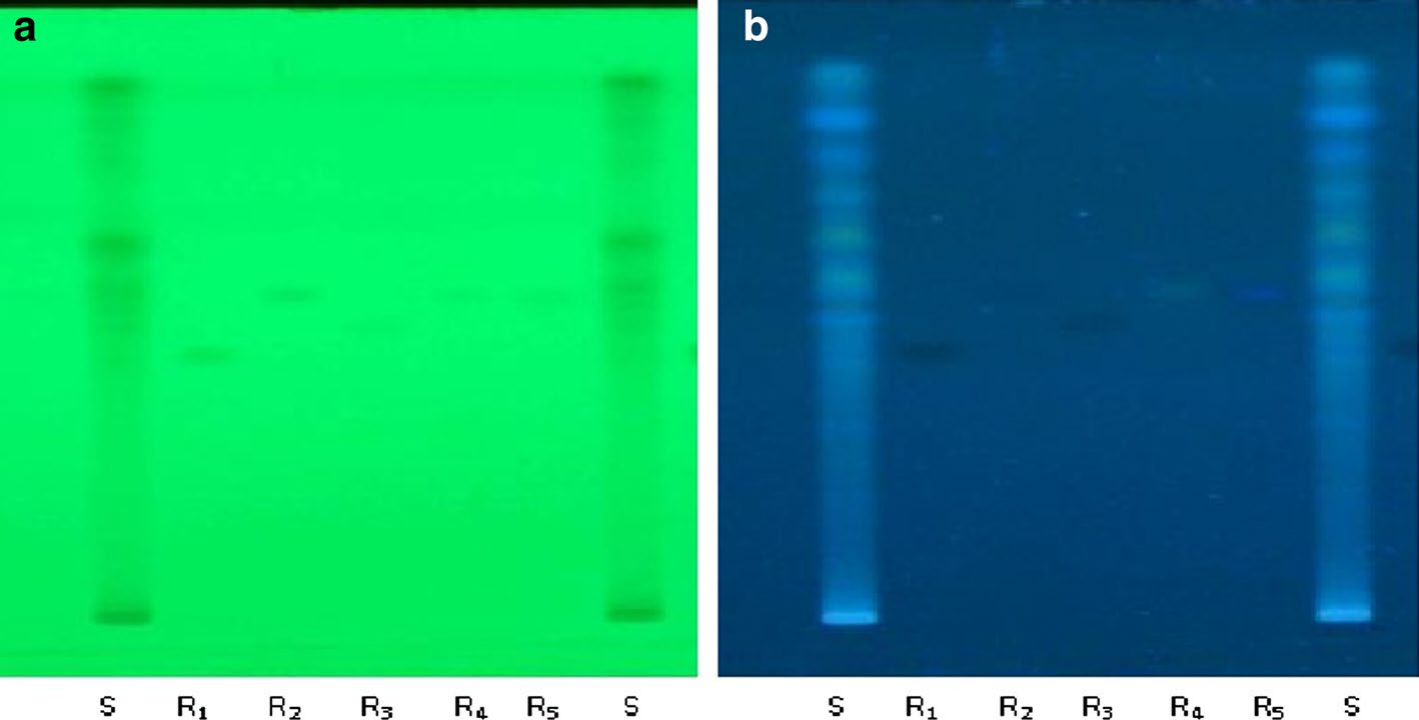
HPTLC chromatogram of standards (*R*_*1*_ protocatechuic acid, *R*_*2*_ vanillic acid, *R*_*3*_ syringic acid, *R*_*4*_ kaempferol and *R*_*5*_ ferulic acid) and sample (*S* methanolic extract) at 254 nm (**a**) and 365 nm (**b**).

**Fig. 2:**
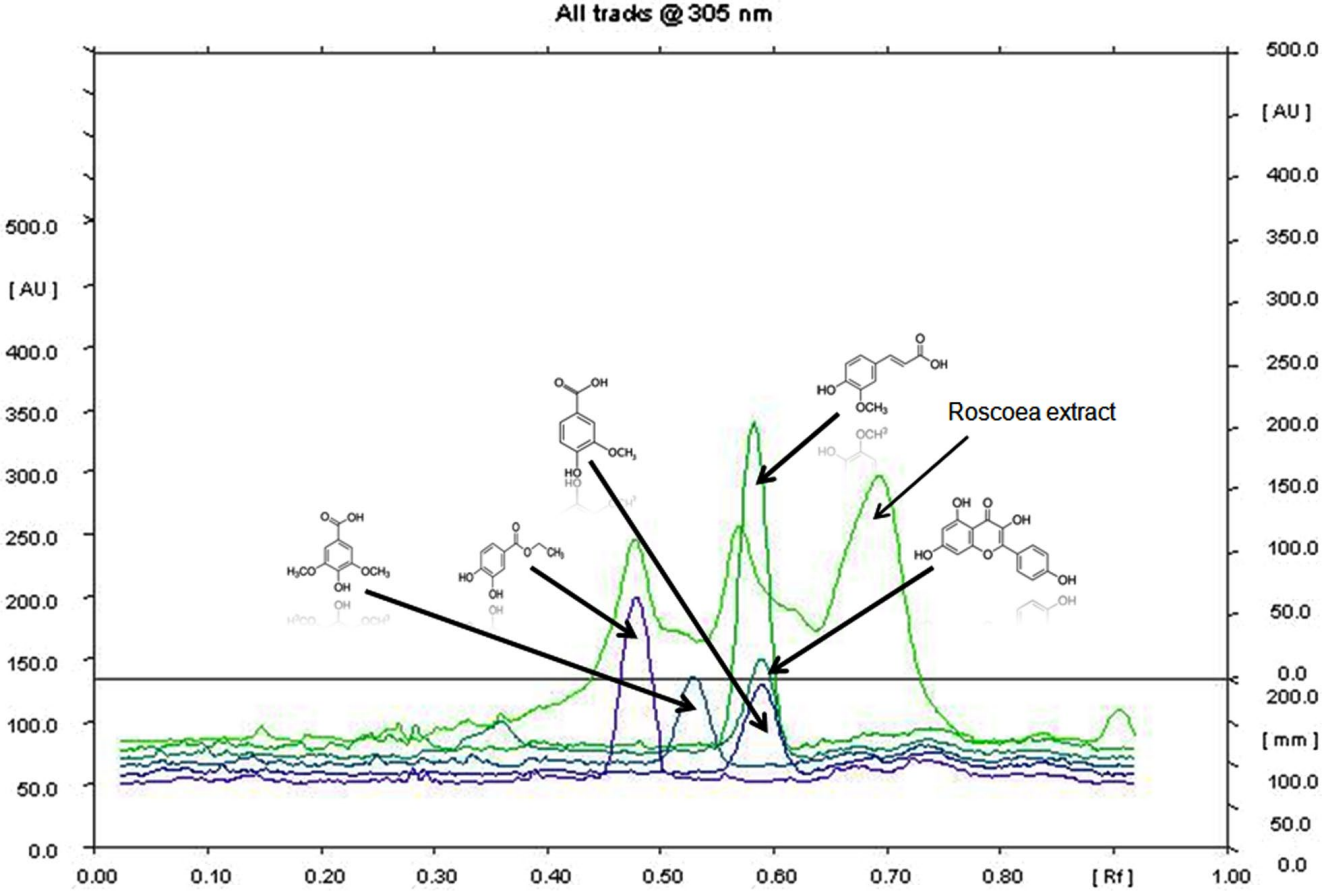
Overlay spectra of standards (*bottom* to *top* in order: protocatechuic acid, vanillic acid, syringic acid, kaempferol and ferulic acid) and *R. purpurea* tubers methanol extract.

Method optimization (Table 3) for chemical profiling of RPE suggested the presence of five major biologically active polyphenolics as protocatechuic acid, syringic acid, vanillic acid, kaempferol and ferulic acid (Figs. 1, 2). Quantification of secondary metabolites reveals that kaempferol (0.30%) was the major metabolite among the other identified markers and then follows the order, vanillic acid (0.27%), protocatechuic (0.14), syringic acid (0.08%) and ferulic acid (0.05%).

**Table 3:** Method validation of chemical markers in *R. purpurea* tubers methanol extract through HPTLC

Parameters	Protocatechuic acid	Vanillic acid	Syringic acid	Kaempferol	Ferullic acid
Linearity range (ng)	100–600	100–600	100–600	100–600	100–600
Regression coefficient (r^2^)	0.993	0.999	0.999	0.999	0.992
LOD (ng)	35	35	35	35	35
LOQ (ng)	100	100	100	100	100
Rf	0.48	0.60	0.53	0.59	0.58
Wavelength (nm)	305	305	285	305	305

(Values are mean, n = 3).

*ng* nanogram.

### Evaluation of anti oxidant potential

**Fig. 3:**
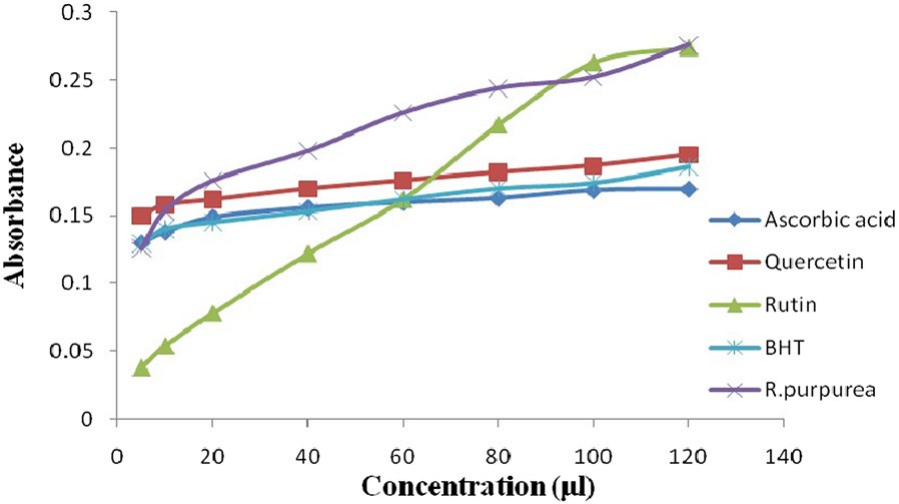
Ferric reducing power assay of standards and *R. purpurea* tubers.

**Fig. 4:**
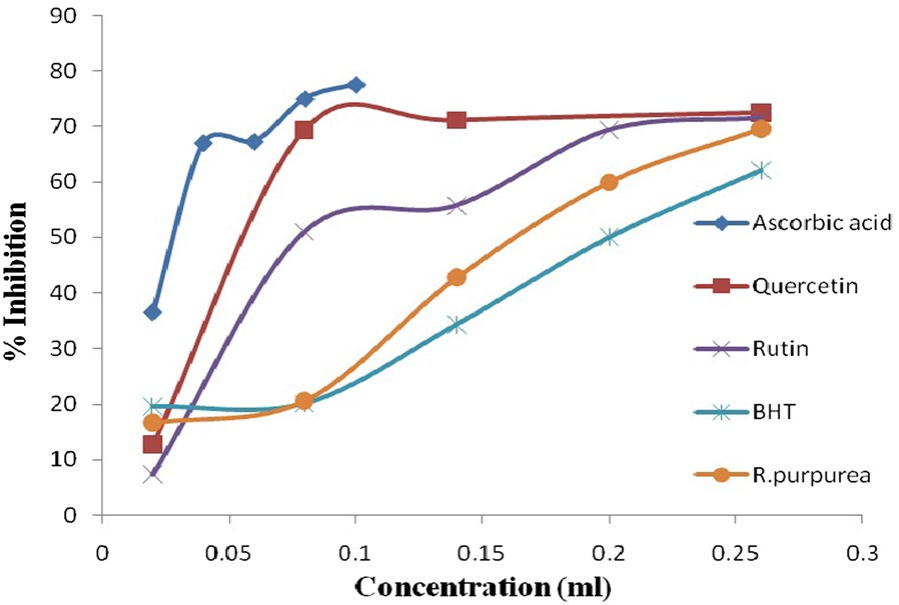
DPPH radical scavenging activity of standards and R*. purpurea* tubers.

**Fig. 5:**
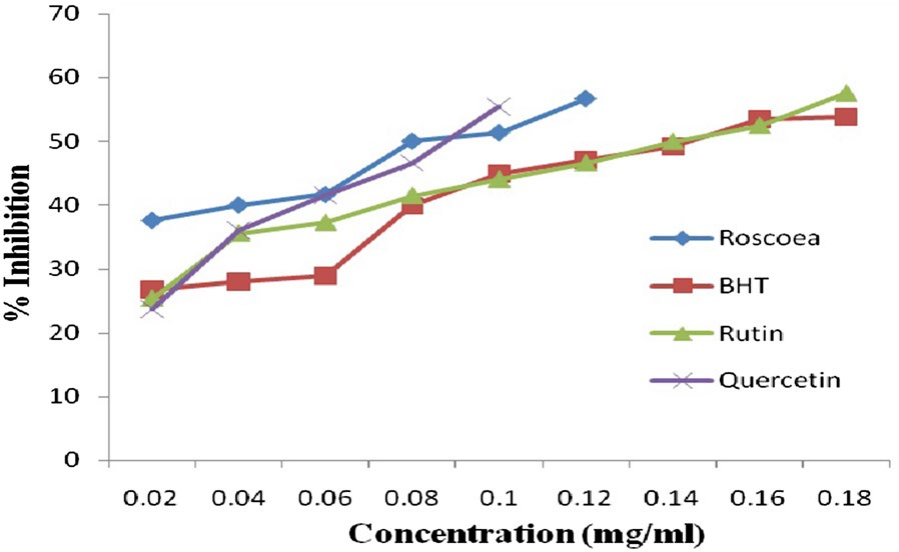
Antioxidant activity of standards and *R. purpurea* tubers as assessed by β-carotene.

Total phenolic and flavonoid content in RPE was 7.1 mg/g GAE and 6.1 mg/g QE as estimated by regression analysis of Gallic acid and quercetin (0.1 mg/ml) as standard (Table 4). The reducing power assay of RPE served as significant indicator of its potentiality as reducing agent, which in turns signifies its anti oxidant activity and data (Fig. 3) reveals that reducing power of RPE increases linearly (r^2^ = 0.946) with increase in concentration, similar to standards i.e. ascorbic acid, quercetin, rutin and BHT respectively. The scavenging effect of DPPH radical was concentration dependant and potentially varied for ascorbic acid, quercetin, rutin and butylated hydroxy toluene (BHT) and RPE (Fig. 4). Ascorbic acid exhibits maximum inhibition of 77.57% which is followed by quercetin, rutin, RPE and BHT having inhibition of 72.43, 71.48, 69.57, 62.10% respectively, although the IC_50_ decreases in order of RPE > rutin > quercetin > ascorbic acid > BHT and hence indicating that BHT is potent inhibitor of free radical in all (Table 5). Antioxidant activity estimated by bleaching of β-carotene for standard viz ascorbic acid, quercetin, rutin and BHT (Fig. 5) were estimated. BHT exhibited IC_50_ at 1.22 mg/ml, thus act as potential anti-oxidant which is followed by quercetin, rutin and RPE (Table 6). Ascorbic acid did not respond to this assay. IC_50_ of RPE, however was significantly different to that of standards when compared at 5 and 1% level of significance (p < 0.01) in both DPPH radical scavenging assay and β-carotene bleaching method. Table 7 reveals that there is positive correlation between IC_50_ value of DPPH and β-carotene beaching method with phenolic and flavonoids content i.e. the antioxidant activity increases linearly with increase in content of phenolics and flavonoids, moreover the values were also significantly (p < 0.01) correlated with each other. Data depicted that correlation is more to flavonoid content (higher r^2^ value) than to phenolic content.

**Table 4:** Polyphenolics content in methanolic extract of *R. purpurea* tuber

S. no.	Total poly phenolics^b^	Values^a^	Regression equation (y)	Regression coefficient (r^2^)
1.	Total phenolics	14.13 ± 0.115	115.9 x + 0.113	0.999
2.	Total flavonoids	12.23 ± 0.055	74.61x + 0.058	0.998

^a^Values are mean ± SD (n = 3).

^b^Total phenolics are represented as mg Gallic acid/gm of dry weight, total flavonoids are represented as mg quercetin/gm of dry weight.

**Table 5:** IC_50_ value of standards and *R. purpurea* in DPPH radical scavenging model

S. no.	Extracts	IC_50_ (µg/ml)*
1.	Ascorbic acid	3.86 ± 0.057
2.	Quercetin	5.93 ± 0.115
3.	Rutin	6.8 ± 0.173
4.	BHT	2.06 ± 0.115
5.	*R. purpurea*	810.66 ± 1.154

* Values are mean ± SD (n = 3), indicating significance at p < 0.01.

**Table 6:** IC_50_ value of standards and *R. purpurea* in β-carotene bleaching method

S. no.	Extracts	IC_50_ (µg/ml)*
1.	Quercetin	8.33 ± 0.115
2.	Rutin	14.06 ± 0.115
3.	BHT	1.22 ± 0.017
4.	*R. purpurea*	600.66 ± 1.154

* Values are mean ± SD (n = 3), indicating significance at p < 0.01.

**Table 7:** Correlations between the IC_50_ values of *R. purpurea* extract in DPPH, β-carotene bleaching method and TPC, TFC content

S. no.	Assay (IC_50_ µg/ml)	Co-relation coefficient (r^2^)*	
		Phenolics	Flavonoids
1.	DPPH assay	0.503	0.986
2.	β-carotene bleeching assay	0.501	0.984

* Indicating significance at p < 0.01.

## Discussion and conclusions

The age old edible use of *R. purpurea* tuber has been validated by results of ongoing study, which reveals the presence of wide range of metabolites/phytochemicals of therapeutic use. Physico chemical standards were also established to identify the authentic species among the other closely related members of genus *Roscoea*. To the best of our knowledge, this is first ever reporting on identification and quantification of chemicals markers (polyphenolics) in *R. purpurea* tuber through HPTLC. The identified markers viz. Protocatechuic acid, syringic acid, vanillic acid, kaempferol and ferulic acid are already well known with validated and potential bioactivities. Polyphenols are the major natural plant inhabiting compounds with anti oxidant activity, which is supposed to be due to their redox potential and thus plays an important role in absorbing, neutralizing, quenching and decomposing the free radical/oxygen and/or superoxide species. Free radical scavenging activity of DPPH is widely used for screening of medicinal plants having anti oxidant activity. The mechanism however, well evident is due to de-colorization of DPPH by electron donated by anti oxidant compound/moiety and thus stabilizing the DPPH radical. BHT is considered as commercial standard because of its wide use in food grade products as anti-oxidant and hence serves as efficient standard to compare with RPE. On the other hand β-carotene bleaching method is based on loss of yellow color of β-carotene, due to its reduction with radicals which are formed by linoleic acid oxidation in emulsion. The rate of bleaching can however be slowed down in presence of anti oxidants and this fact is used in evaluation of anti oxidant activity of extract in comparison with natural and synthetic standards. In spite of the fact that polar compound ascorbic acid is well known antioxidant, it does not respond to β-carotene bleaching model. This interesting phenomenon is formulated as “polar paradox” and has been reported earlier by several other workers also [19–21] that the polar antioxidants remaining in the aqueous phase of the emulsion are more diluted in lipid phase and are thus less effective in protecting the linoleic acid.

In a conclusion the present studies establish the physicochemical standards of *R. purpurea* which is essential for identification and quality evaluation of one of the main ingredient herb of “Ashtavarga”, a polyherbal Ayurvedic formulation. Quantification of metabolites suggested that the tubers are nutritionally rich having appreciable content of fiber (28.1%), protein (3.46%) and oil (3.5%). Presence of poly phenolics content viz. TPC (14.13 mg/gm GAE) and TFC (12.23 mg/gm QE) was further confirmed by identification and quantification through HPTLC. The anti oxidant potential of *R. purpurea*, as estimated by three models having different mechanism of action suggested the potential activity in species, although IC_50_ varies within assay’s. In addition to this, there exists a positive, significant correlation between the phenolic (r^2^ more than 0.500) and flavonoids (r^2^ more than 0.900) content with the anti oxidant activity of *Roscoea* extract. This will aid in standardization for quality, purity and sample identification with the presence of various therapeutically/nutritionally active chemical markers along with potential bioactivity. Hence, the study supports the fact that tubers of *R. purpurea* had good nutritional quality with promising antioxidant activity. These results suggested that *Roscoea* contain biologically/therapeutically active compounds, however to justify these claims we need to do more extensive study based on activity guided fractionation in the future.
